# Benchmarking Precompensated Current-Modulated Diode-Laser-Based Differential Absorption Lidar for CO_2_ Gas Concentration Measurements at kHz Rate

**DOI:** 10.3390/s25196064

**Published:** 2025-10-02

**Authors:** Giacomo Zanetti, Peter John Rodrigo, Henning Engelbrecht Larsen, Christian Pedersen

**Affiliations:** Department of Electrical and Photonics Engineering (DTU Electro), Technical University of Denmark, Frederiksborgvej 399, Building 128, 4000 Roskilde, Denmark; pejr@dtu.dk (P.J.R.); heel@dtu.dk (H.E.L.); chrp@dtu.dk (C.P.)

**Keywords:** differential absorption lidar, CO_2_ remote sensing, gas spectroscopy, tunable diode laser absorption spectroscopy

## Abstract

We present a tunable diode-laser absorption spectroscopy (TDLAS) system operating at 1.5711 µm for CO_2_ gas concentration measurements. The system can operate in either a traditional direct-mode (dTDLAS) sawtooth wavelength scan or a recently demonstrated wavelength-toggled single laser differential-absorption lidar (WTSL-DIAL) mode using precompensated current pulses. The use of such precompensated pulses offsets the slow thermal constants of the diode laser, leading to fast toggling between ON and OFF-resonance wavelengths. A short measurement time is indeed pivotal for atmospheric sensing, where ambient factors, such as turbulence or mechanical vibrations, would otherwise deteriorate sensitivity, precision and accuracy. Having a system able to operate in both modes allows us to benchmark the novel experimental procedure against the well-established dTDLAS method. The theory behind the new WTSL-DIAL method is also expanded to include the periodicity of the current modulation, fundamental for the calculation of the OFF-resonance wavelength. A two-detector scheme is chosen to suppress the influence of laser intensity fluctuations in time (1/f noise), and its performance is eventually benchmarked against a one-detector approach. The main difference between dTDLAS and WTSL-DIAL, in terms of signal processing, lies in the fact that while the former requires time-consuming data processing, which limits the maximum update rate of the instrument, the latter allows for computationally simpler and faster concentration readings. To compare other performance metrics, the update rate was kept at 2 kHz for both methods. To analyze the dTDLAS data, a four-parameter Lorentzian fit was performed, where the fitting function comprised the six main neighboring absorption lines centered around 1.5711 µm. Similarly, the spectral overlap between the same lines was considered when analyzing the WTSL-DIAL data in real time. Our investigation shows that, for the studied time intervals, the WTSL-DIAL approach is 3.65 ± 0.04 times more precise; however, the dTDLAS-derived CO_2_ concentration measurements are less subject to systematic errors, in particular pressure-induced ones. The experimental results are accompanied by a thorough explanation and discussion of the models used, as well as their advantages and limitations.

## 1. Introduction

Tunable diode laser absorption spectroscopy (TDLAS) is a differential laser absorption spectroscopy technique that exploits the wavelength tunability of diode lasers to probe the properties of the medium wherein the laser propagates. In the context of gas spectroscopy, the main purpose of TDLAS is gas species quantification and leak detection [1,2,3,4,5,6,7,8]. The realization of TDLAS systems and their operation can be quite diverse and is highly dependent on the application. Even when it comes to environmental monitoring, the design of the apparatus can be very different if the aim is to monitor a ubiquitous gas (e.g., CO_2_ concentration in the atmosphere), or if spatially resolved quantification is needed (e.g., mapping CO_2_ concentration over an area with localized emitters, such as farms or industries). In this paper, we will focus on the latter scenario, namely the quantization of CO_2_ concentrations in settings where remote probing of spatially separated locations must be performed by the same system. In these conditions, a widespread paradigm is that of a direct linear wavelength scan (dTDLAS). This approach requires a diode laser’s wavelength to be periodically modulated over an absorption line by driving the laser with a sawtooth current profile. The measured absorbance is then fitted with a model line shape, some parameters of which depend on the target quantity to measure (typically concentration), and the target metric is eventually extracted from such parameters. This method has many advantages, such as the possibility of being calibration-free and allowing the quantification of multiple gases whose absorption lines lie within the scanned wavelength window. On the other hand, fitting algorithms strongly limit the speed of data acquisition when high-speed analysis is required, therefore many systems that employ this architecture rely on data post-processing to refine raw data and extract more accurate results [9]. Another paradigm, firstly demonstrated in 1966, is differential absorption lidar (DIAL) [10,11,12,13,14]. In this configuration, the gas concentration is calculated from the measured transmittance at two different wavelengths, generated by two separate lasers [13] or a single dual-wavelength laser [14]. Recently, a particular and more optimized version of the single laser DIAL relying on a simple tunable diode laser [15] has emerged. This technique utilizes the center of an absorption line as first wavelength whereas the second one is selected far from it, relative to the width of the gas line, and digital controls are put into place to ensure locking of the laser on the absorption line over extended periods of time. Additionally, what makes it stand out is the use of a single tunable diode laser that is controlled using a precompensated current modulation, which allows for precise wavelength transitions on a timescale approximately two orders of magnitude shorter than dictated by the slow thermal constants of the laser’s response [16], thus allowing for kHz update rates. We will refer to this as the wavelength-toggled single laser differential-absorption lidar (WTSL-DIAL) method. Similar to the scanning method, WTSL-DIAL can be calibration-free but is limited to the quantification of a single gas species. The quantification of more than one gas species requires extending the two-wavelength approach to toggling between three or more distinct wavelengths. When it comes to concentration measurement update rate, however, the much simpler data processing of the WTSL-DIAL approach essentially removes any computational speed limitations, which are instead particularly relevant in dTDLAS. Streamlining computations can be translated into much faster real-time concentration measurements, which not only reduce the noise coming from laser intensity fluctuations (pink noise) but also limit the influence of mechanical vibrations on the measured quantities. On top of this, a high update rate instrument can be combined with a rapidly scanning device to realize a system able to capture the dynamics of targeted gaseous species over extended areas, pivotal in some applications such as leak detection and warfare.

In this paper, we will expand on the theory of the novel WTSL-DIAL approach, including the periodicity of the signal, and systematically compare its performance against the dTDLAS approach to gauge the new method’s standing among other well-established techniques. To achieve this, the same experimental setup is used to compare the two methods in the case of CO_2_ quantification at 2 kHz. This update rate was chosen because it is close to the fastest real-time analysis speed currently achievable by wavelength scanning fitting methods [17]. Target metrics of interest are precision, repeatability and stability over time, while particular attention is given to the concept of accuracy and calibration. In fact, while scientific articles typically analyze spectroscopic techniques in relative terms, for example through signal-to-noise ratio (SNR) estimations or through measurements of relative stability over time, particular attention must also be paid to the uncertainty originating from the theoretical models used and to the approximations made. Therefore, here both aspects are explored in detail for the two spectroscopic techniques in question. To accompany the experimental results and considerations, a sensitivity analysis is conducted by simulating the setup.

## 2. Theoretical Background

The physical phenomenon that TDLAS exploits is the ability of gases to absorb radiation at extremely specific optical frequencies, namely those that allow the transition between two non-degenerate rovibrational states. While such transitions can be derived from first principles, a more pragmatic approach is to rely on libraries that contain a vast and detailed set of transitional parameters that have been experimentally derived and allow the analytical computation of absorption lines under different environmental conditions. In this paper we use one of such libraries, HITRAN2020 [18], to calculate various parameters, and it also serves as the backbone of our simulations. However, the tabulated data in this library comes (at least partially) from empirical data and is hence subject to error, which imposes a lower limit on the precision of the experiments that rely on it. A Monte-Carlo sensitivity analysis of our setup at a typical working temperature of 295.12 K shows that the relative error induced by the uncertainty on the tabulated data is in the order of 2.25% for dTDLAS and 2.11% for WTSL-DIAL. These are quite limiting because they greatly exceed the stability of our system. Therefore, when combining sources of error, our experimental uncertainties would be overshadowed by their counterpart on the tabulated data. For this reason, in the rest of the paper, the uncertainty on the HITRAN2020 tabulated values is considered separately from our experimental error, although both must be taken into account when calculating the total uncertainty. This aspect will be brought up when presenting the results and comparing the two approaches, as one could be more susceptible to the problem than the other.

### 2.1. Spectral Line Shape and Approximations

As described in [18], the absorbance of gases is typically considered to take the shape of a Voigt function in the frequency domain, which is the result of the convolution between a Lorentzian and a Gaussian curve, representing the pressure and Doppler broadening of the transition, respectively. Using approximated formulas to estimate Voigt’s full width at half maximum (FWHM), known as pseudo-Voigt approximations [19], it can be shown that in standard conditions, on the Earth’s surface, the contribution of Lorentzian broadening for the CO_2_ transition at 1.5711 µm is about 180 times greater than the Gaussian one. Similarly, Voigt’s FWHM is accurately described by the Lorentzian FWHM with a relative residue of less than 0.8%. Under these conditions, it is here reasonable to neglect Doppler broadening, hence all the absorption lines are considered to be purely Lorentzian in what follows.

Another approximation comes from considering the Lorentzian line shape in the wavelength domain, instead of the frequency domain. The reason for using the wavelength domain is very practical since the output wavelength of the diode laser is linear with the current above the lasing threshold. In mathematical terms, this approximation corresponds to allowing the following equality to hold:

(1) $$\frac{1}{1+{\left|\frac{v\;-\;v_{0}}{∆v}\right|}^{2}}\approx \frac{1}{1+{\left|\frac{\lambda \;-\;\lambda_{0}}{∆\lambda }\right|}^{2}}$$

where v is the frequency of the laser, $v_{0}$ the central frequency of the transition, ∆v is its half width at half maximum (HWHM), $\lambda =\frac{c}{v}$ and $\lambda_{0}=\frac{c}{v_{0}}$, with c being the speed of light in vacuum (we also assume the refractive index of the gas to be 1). If we define $v_{1}$ and $v_{2}$ so that $∆v=v_{1}-v_{2}$, then $∆\lambda =\lambda_{2}-\lambda_{1}$, with $\lambda_{1,2}=\frac{c}{v_{1,2}}$. It is easy to show that this approximation is better when the ratio $\frac{\lambda_{1}\lambda_{2}}{\lambda \lambda_{0}}$ is close to 1, which, at worst, is about 2 × 10^−4^ away from 1 for the wavelength window explored. Such an error is much smaller than the relative error on the HITRAN2020 halfwidth parameters for this transition (between 2% and 5%), and we will therefore use the approximation.

Finally, the last approximation is to calculate the HWHM in the wavelength domain as $∆\lambda =c\frac{∆v}{v_{0}^{2}}$ that comes with a relative error of $\frac{{∆v}^{2}}{v_{0}^{2}}$, which is quite negligible (2 × 10^−10^).

In the experimental conditions of this work, the combination of the sources of error coming from working in the wavelength domain results in a total relative error of about 1 × 10^−5^ in the conversion from raw data to concentration values.

### 2.2. Data Processing: dTDLAS

In dTDLAS, the current is periodically modulated in a sawtooth fashion. By performing an impulse response study analogous to the one presented in [15], but now accounting for the periodicity of the signal (see Supplementary Materials for the detailed theoretical description), the wavelength modulation over time arising from the current modulation can be calculated. Translating the time axis into a wavelength axis then allows for the fitting of the absorption line, which will be considered as having a Lorentzian functional form in the wavelength domain.

To derive the absorption line, the raw data (in our case, the transmittance of the laser) is converted into absorbance by assuming the validity of Lambert–Beer’s law of absorption. Then, we perform a Lorentzian fit with four parameters, *A*, *B*, *C* and *D*, so that the fitting function is

(2) $$\frac{\frac{A}{C}}{1+{\left|\frac{t\;-\;B}{C}\right|}^{2}}+D$$

where t denotes time; hence, the transition to the wavelength domain has not been made yet. This means that *B* and *C* have temporal units. Additionally, this requires the “baseline”, namely the ratio of the two detectors’ readings over the spanned wavelength range, to be flat, as it is fitted by the constant *D*.

**Figure 1:**
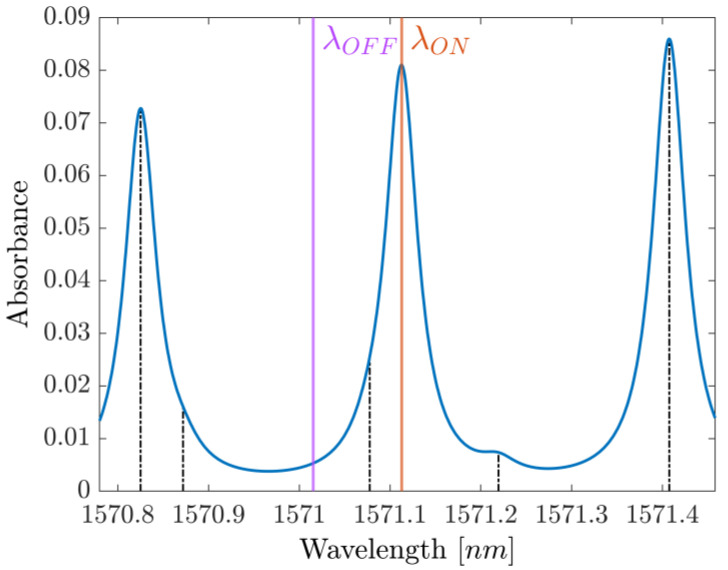
Absorbance profile around the probed spectrum. ON- and OFF-resonance wavelengths are labeled as $\lambda_{ON}$ and $\lambda_{OFF}$, respectively, while the position of the other five main absorption lines is indicated in black.

The results of this first fit are then used as initial parameters for a more sophisticated Lorentzian fit, which includes the six strongest CO_2_ lines around the working point of the laser, the fourth of which is the main absorption line we are targeting, as shown in Figure 1. This second fit is still a four-parameter fit, where the *A_i_*, *B_i_* and *C_i_* parameters (*i* = 1, 2, 3, 4, 5, 6) are expressed in terms of the main line’s parameters (*i* = 4) using the tabulated HITRAN values as coefficients. The fitting function is then

(3) $$D+\sum_{i=1}^{6}\frac{\frac{A_{i}}{C_{i}}}{1+{\left|\frac{t-B_{i}}{C_{i}}\right|}^{2}}=D+\sum_{i=1}^{6}\frac{\frac{a_{i}A_{4}}{{c_{i}C}_{4}}}{1+{\left|\frac{t-\left(B_{4}+b_{i}C_{4}\right)}{c_{i}C_{4}}\right|}^{2}}=D+\sum_{i=1}^{6}\frac{\frac{a_{i}A}{c_{i}C}}{1+{\left|\frac{t-\left(B+b_{i}C\right)}{c_{i}C}\right|}^{2}}$$

so that the parameters of interest, *A*_4_, *B*_4_ and *C*_4_ were re-labeled as *A*, *B* and *C* to clarify that those, in addition to *D*, are the only parameters of the fitting procedure. The introduced *a_i_*, *b_i_* and *c_i_* constants are defined as follows:

(4) $$\begin{array}{l} a_{i}=\frac{S_{i}}{S_{4}}\frac{\lambda_{i}^{2}}{\lambda_{4}^{2}}\\ b_{i}=\frac{\lambda_{i}-\lambda_{4}}{\gamma_{4}}\;\;\\ c_{i}=\frac{\gamma_{i}}{\gamma_{4}} \end{array}$$

where $\lambda_{i}$ is the wavelength of the *i*th line, $\gamma_{i}$ the HWHM of the *i*th line in the wavelength domain, and *S_i_* is the line intensity, as defined in the HITRAN documentation. All of these quantities must be calculated for the actual temperature and pressure conditions of the experiment.

When it comes to the interpretation of the fit results, the aim here is to extract concentration; thus, the interest is on the *A* parameter. It can be read as

(5) $$A=\frac{nLS_{4}\lambda_{4}^{2}}{\pi k}$$

where n is the concentration, L the path length of the laser beam through the region where the gas is present, and k is the conversion factor from time to wavelength. Note that this derivation relies on the same choice of units as in HITRAN, and that it assumes a uniform distribution of the gas throughout L. Once the fit is obtained, the concentration is derived by inverting Equation (5).

### 2.3. Data Processing: WTSL-DIAL

In the case of WTSL-DIAL, we are assuming that the underlying transmittance profile arises from the same six absorption lines considered in the wavelength scanning method. This time, however, the width, position and intensity of the peaks are not fitting parameters but are instead calculated from the tabulated line parameters for the temperature and pressure conditions of the experiment. Therefore, in the wavelength domain, the absorbance is

(6) $$\alpha \left(\lambda \right)=nL\sum_{i=1}^{6}\frac{S_{i}\lambda_{i}^{2}}{\pi \gamma_{i}}\frac{1}{1+{\left|\frac{\lambda \;-\;\lambda_{i}}{\gamma_{i}}\right|}^{2}}$$

where the terms are the same as in dTDLAS, again expressed in the units used in HITRAN.

By exploiting the impulse response of the system, one can calculate the wavelength modulation when applying any kind of current modulation, as long as the system can be considered linear and time-invariant. This makes it possible to experimentally compensate for the slow wavelength response of the diode laser by optimizing the current modulation to have a fast and stable transition between two wavelengths. One of them coincides with the *i* = 4 resonance (ON), while the other is substantially free of absorption (OFF). Knowing the ON and OFF wavelengths, it is possible to calculate $\alpha_{ON}$, by excluding *i* = 4, which we label as $\alpha_{ON}^{i\neq 4}$, as well as $\alpha_{OFF}$.

Assuming the validity of Lambert–Beer’s law, the negative logarithm of the ratio between ON and OFF transmittance data provides an experimental estimation of $\alpha_{ON}-\alpha_{OFF}$, namely $\alpha_{ON,exp}-\alpha_{OFF,exp}$. Therefore,

(7) $$-\ln\left(\frac{T_{ON}}{T_{OFF}}\right)=\alpha_{ON,exp}-\alpha_{OFF,exp}$$

where T denotes transmittance. Isolating the contribution of the *i* = 4 line to the ON absorbance, $\alpha_{ON,exp}^{i=4}$, from Equation (7) leads to

(8) $$-\ln\left(\frac{T_{ON}}{T_{OFF}}\right)=\alpha_{ON,exp}^{i=4}+\alpha_{ON,exp}^{i\neq 4}-\alpha_{OFF,exp}=\alpha_{ON,exp}^{i=4}\left(1+\frac{\alpha_{ON,exp}^{i\neq 4}}{\alpha_{ON,exp}^{i=4}}-\frac{\alpha_{OFF,exp}}{\alpha_{ON,exp}^{i=4}}\right)$$

Now, the quantity in the parentheses on the rightmost side of Equation (8) does not depend explicitly on concentration nor does it depend on the optical path length L, as all absorbances are ratioed, although concentration enters implicitly through the partial pressure that determines the $\gamma_{i}$. If we momentarily disregard this implicit dependency, we can use the measured temperature and pressure values to estimate the quantity in the parentheses using the HITRAN2020 library. After that, rearranging the equation allows the immediate extraction of the concentration n from $\alpha_{ON,exp}^{i=4}$.

As previously stated, this method relies on the correctness of the calculated quantities, and some of these quantities in turn depend on the concentration. Although the impact of this dependency is somewhat limited due to the fact that $\alpha_{ON}^{i=4}\gg \alpha_{ON}^{i\neq 4}$ and $\alpha_{ON}\gg \alpha_{OFF}$, it becomes, however, relevant when showing results or when defining a data acquisition protocol.

## 3. Experimental Setup

The setup consists of a 1.571 µm diode laser (EP1572-5-NLW-B26-100FM, Eblana Photonics, Dublin, Ireland), which is fiber-coupled to a polarization-maintaining fiber of about 1 m in length, terminated with a beam collimator (F220FC-1550, Thorlabs Inc., Newton, NJ, USA). The laser is driven by a laser diode current controller (LDC205C, Thorlabs Inc., Newton, NJ, USA), while its temperature is regulated by a temperature controller (TED 200, Thorlabs Inc., Newton, NJ, USA) set to 25.00 °C. This temperature allows us to probe the CO_2_ line centered at 1.5711 µm by driving the laser with a current of about 225 mA.

**Figure 2:**
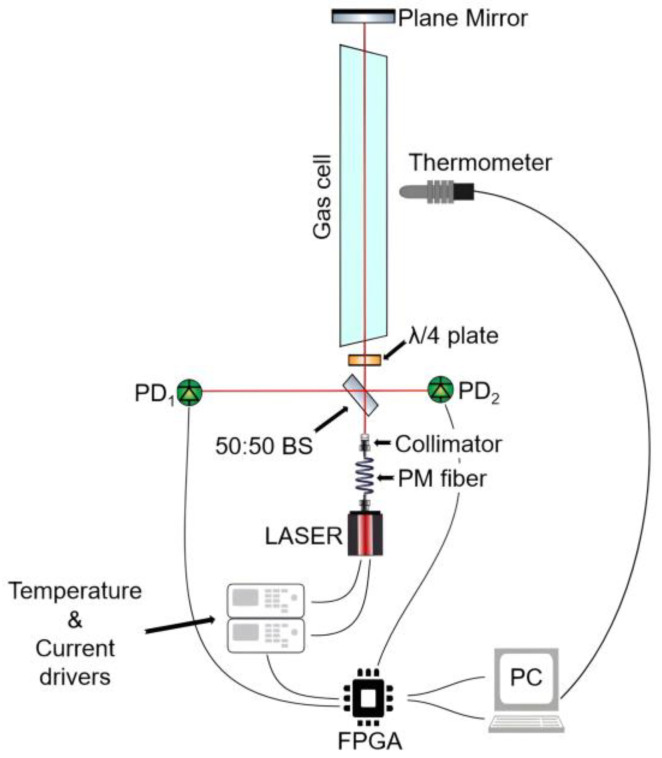
Sketch of the setup. The green circles represent the photodetectors (PD), namely PD_1_ acting as reference and PD_2_ as the gas arm.

After the collimator, the laser beam propagates in free space through a 50:50 non-polarizing plate beamsplitter (10B20NP.31, Newport Corporation, Irvine, CA, USA), after which one beam is detected by a first InGaAs photodiode detector (FGA21, Thorlabs Inc., Newton, NJ, USA), which acts as reference (PD_1_ in Figure 2). The second beam passes a λ/4 achromatic waveplate (A-12.7-A-.250-B-3, Edmund Optics Inc., Barrington, NJ, USA) which limits the interference with unwanted stray reflections coming from the other optical components. The beam then passes through a 40 cm custom-made gas cell (Wavelength References Inc., Corvallis, OR, USA) filled with a mixture of C_2_H_2_/CH_4_/CO_2_/N_2_. The nominal volume mixing ratio (VMR) is 1% C_2_H_2_, 3% CH_4_, 80% CO_2_, balanced with N_2_ to 740 Torr total pressure. The gas cell has tilted, wedged windows made of B270 glass, AR-coated for 1550 nm. A plane mirror is used to make the laser beam trace back to the beamsplitter, which then directs part of the radiation towards a second photodetector (PD_2_ in Figure 2, nominally identical to PD_1_), serving as the gas arm. While the same beamsplitter is used, the return beam does not fully overlap with the transmitted one, as this, together with the λ/4 waveplate, highly reduces interferences coming from undesired reflections. Moreover, the rotation angle of the waveplate can be adjusted to make the observed absorbance baseline flat.

As shown in Figure 2, most of the electronic components are controlled by a field-programmable gate array (FPGA) embedded in a controller (cRIO-9063, National Instruments, Austin, TX, USA), which mounts a digital-to-analog converter (NI-9263, National Instruments, Austin, TX, USA) and an analog-to-digital converter (NI-9223, National Instruments, Austin, TX, USA). The 128 kSamples/s sampling rate, combined with 64 points per period, determines the 2 kHz update rate. Lastly, a laptop computer (EliteBook 840 G10, HP Inc., Palo Alto, CA, USA) is used to retrieve and process the FPGA readings through a LabVIEW (version 2024 Q3 64-bit) self-developed code, as well as to control a temperature sensor (TSP01, Thorlabs Inc., Newton, NJ, USA) positioned in the gas cell’s proximity, whose output is read every 3 s. The gas cell is assumed to be at thermal equilibrium with the room.

It is important to note that, in both dTDLAS and WTSL-DIAL, data analysis is performed using transmittance; hence, the PD_2_ readings are normalized by the simultaneous PD_1_ readings.

## 4. Operation and Results

### 4.1. dTDLAS

**Figure 3:**
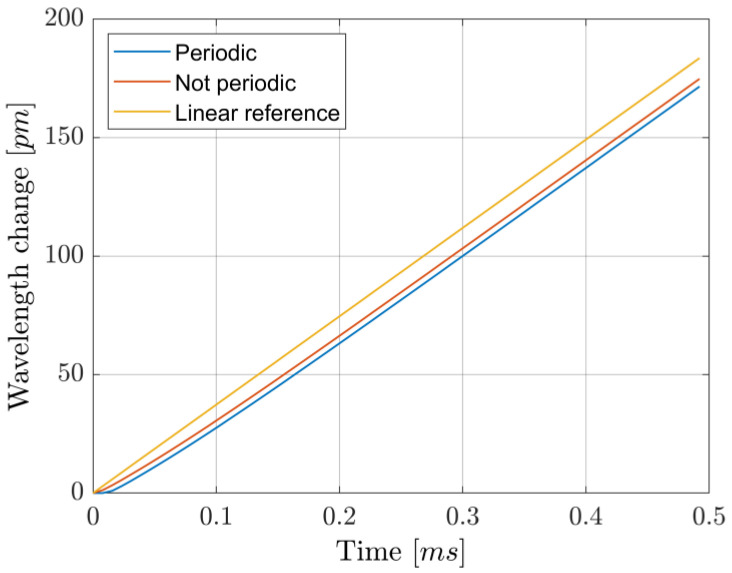
Simulated wavelength response over one period when using a sawtooth current profile at 2 kHz. The difference between the analysis of the signal where periodicity is accounted for or not is observed as a ~2% difference in the scanned range. The “Linear reference” line shows the simulated wavelength modulation in the ideal case where the system responded instantly to changes in the driving current.

**Figure 4:**
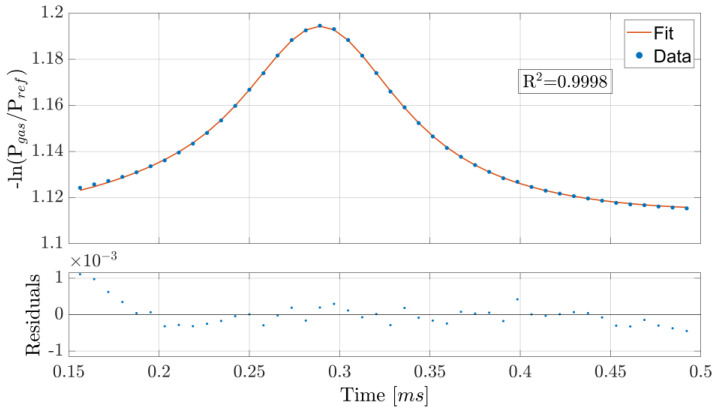
Example of scanned wavelength acquisition and Lorentzian fit. Only the 44 datapoints used for fitting are shown, and the initial ones deviate more from the fit probably due to the slight nonlinearity of the wavelength scan in time, as shown in Figure 3.

The wavelength was periodically scanned across the 1.5711 µm absorption line at a rate of 2 kHz. Within a single period, 64 datapoints were acquired, but only 44 were used for fitting: the periodicity of the signal influences the shape of the wavelength modulation over time, which is not a perfectly linear ramp, as shown in Figure 3. It is possible to compensate for this modulation distortion originating from the finite diode laser wavelength response time by changing the shape of the current modulation. However, such a procedure is highly prone to error due to having to heavily rely on simulation results, unless additional components are added to monitor the actual instantaneous wavelength. We instead opted to neglect the initial datapoints and consider only those where a linear wavelength behavior is reached, which leaves us with a ~69% data usage efficiency. This lowers the reliance on simulations. In addition, including the periodicity of the signal in the simulations can be important, as it results in a ~2% difference in the estimation of the scanned wavelength range, at least under our experimental conditions (see Figure 3). Despite this, both show good linearity (less than 1% difference in slope from the ideal instantaneously responding system) after the same amount of time, here after the initial 23% of the period. Due to the high computational requirements of the fitting procedure, data analysis was performed only after acquisition. Least-squares fitting was performed, with the Levenberg–Marquardt algorithm. An example of fitted data is shown in Figure 4.

**Figure 5:**
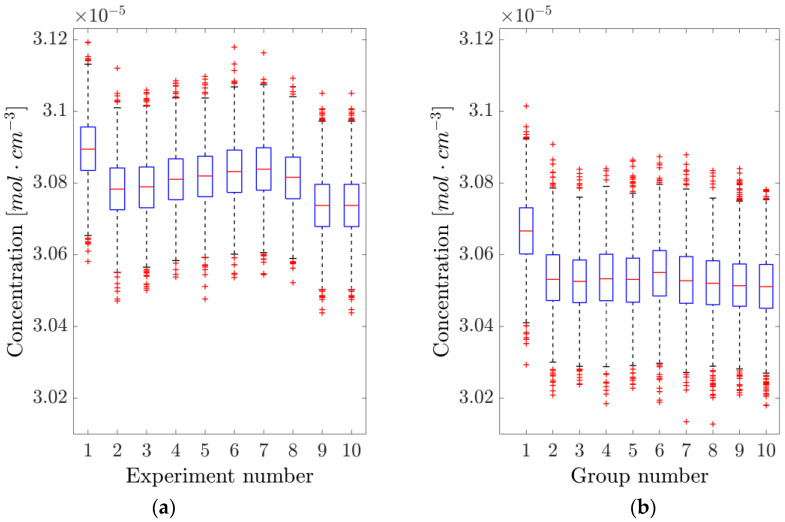
Analysis of the repeatability of the direct tunable diode laser absorption spectroscopy (dTDLAS) method. (**a**) The concentration is measured by resetting the system and acquiring 1 s of data. (**b**) The system is left running, and data is acquired every 2 min. The blue boxes represent the 25th and 75th percentile, the orange horizontal lines are the medians, black whiskers represent the 99.3% confidence interval, while the red crosses indicate outliers.

Multiple 1 s acquisitions, corresponding to 2000 adjacent concentration readings each, were performed. Each time a new experiment was started, the laser was brought below threshold, and the LabVIEW program restarted. The data were collected after waiting for the laser to settle back around its working point. The whole procedure was timed so that an experiment was conducted every 2 min. Although not giving compatible results, the maximum absolute relative difference among the averages of the subsequent experiments was 0.37% (mean 0.10 ± 0.04%). These results are portrayed in Figure 5a.

On the other hand, acquiring 1 s of data every 2 min, without switching off the laser or restarting the LabVIEW code, yielded the results shown in Figure 5b. In this case, compatibility is still not achieved, and the maximum absolute relative difference among the averages of the different experiments was 0.42% (mean 0.08 ± 0.04%), compatible with the with-reset case, hence suggesting that restarting the system has no relevant effect on the scanning approach.

**Figure 6:**
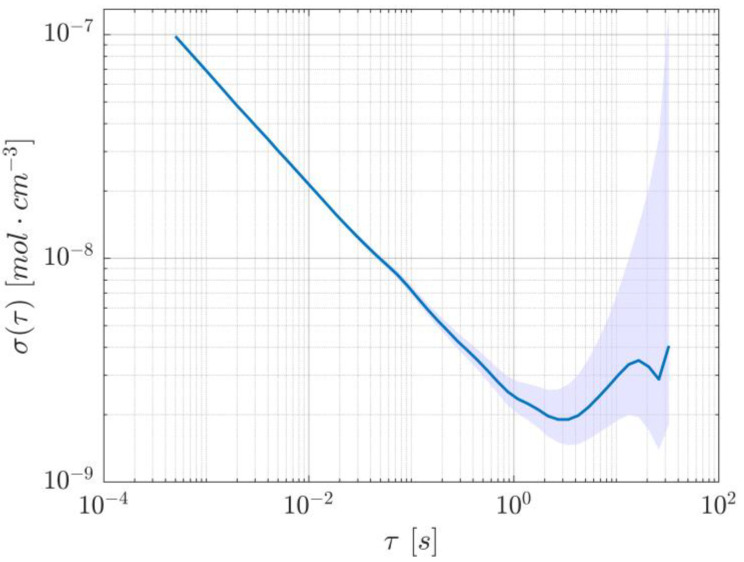
Allan deviation of the dTDLAS method, showing a minimum at about 4.1 s. The shaded area represents the 95% confidence interval.

Lastly, to measure the system’s noise as a function of averaging time, a slightly longer acquisition was performed to calculate the overlapping Allan deviation, which is illustrated in Figure 6. Since the datapoints with the lowest precision lie in the region where flicker noise dominates, the 95% confidence interval was calculated using the empirical number of degrees of freedom found in [20] for flicker noise in frequency modulated-like data.

The most relevant figures of merit for the dTDLAS technique are reported in Table 1.

### 4.2. WTSL-DIAL

**Figure 7:**
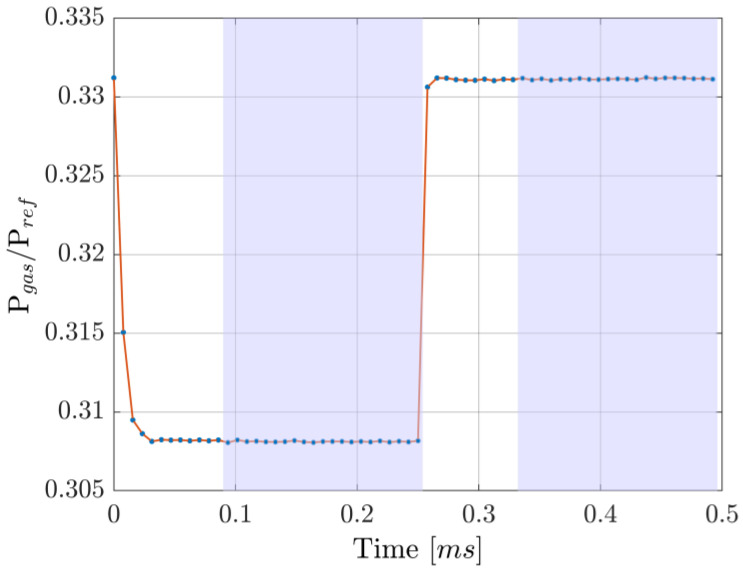
Example of a wavelength-toggled single laser differential-absorption lidar (WTSL-DIAL) acquisition. The datapoints used to calculate the concentration are those lying in the purple shaded regions: ON-resonance ones to the left and OFF-resonance ones to the right. The orange line only serves as a visual guide.

**Figure 8:**
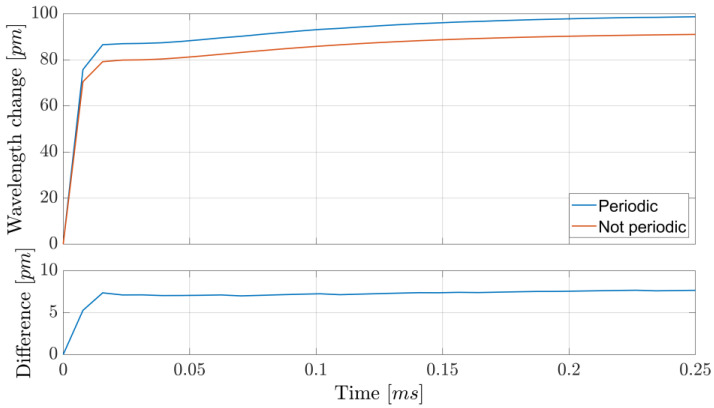
Simulated wavelength response to the current modulation used experimentally. While qualitatively similar, the two simulations exhibit an almost constant separation of about 7.5 pm, highlighting the importance of considering the periodicity of the signal when calculating the OFF-resonance wavelength.

Wavelength toggling was achieved by applying a train of precompensated current pulses. Following the procedure reported in [15], a decaying single exponential was added to the squared current modulation. The amplitude and timescale of the exponential part were experimentally optimized to achieve a fast transition between two wavelengths, as shown in Figure 7, one of which was kept on the 1.5711 µm resonance by a hill-descent algorithm working on the ON-resonance transmittance readings at an update time of 100 ms. In detail, the algorithm involved changing the current in 5 µA steps (positive or negative) and looking at the measured change in the ON transmittance, low-pass filtered at 300 Hz: if the current change caused the transmittance to increase, the next current step was taken with the opposite sign; otherwise, it remained the same. To limit the effect of small fluctuations in the photodiodes’ readings, happening in a timescale of about 1 s, a threshold of 10 same-sign steps was set, after which the step direction was reversed. Note that, due to the non-stationarity of the system over long periods of time and temperature/pressure changes, an algorithm without definitive stopping criteria was necessary. Out of the 64 points of a full period, 21 were used as ON-resonance transmittance measurements and another 21 as OFF-resonance, leading to a ~66% data usage efficiency. Simulations were performed to find the OFF-resonance wavelength, which is needed to compute the OFF terms in Equation (8). Contrary to the dTDLAS method, where the rate of change in wavelength is the important parameter to know in order to extract concentrations (cfr. k term in Equation (5)), in the WTSL-DIAL approach it is the wavelength span induced by the modulation. Therefore, the periodicity of the signal is much more relevant in this context, as can be seen from the difference in the wavelength scan ranges shown in Figure 8. As for the dependency on the guessed partial pressure used to compute Equation (8), the fact that $\alpha_{ON}\gg \alpha_{OFF}$ limits the impact of incorrect estimations of the spanned wavelength range on the final estimation of the concentration.

While we relied on post-processing to analyze the dTDLAS data, the hardware we used allowed for real-time concentration measurements at 2 kHz using the WTSL-DIAL approach. Apart from the advantage of sensing concentration values in real time, online partial pressure estimations (using the ideal-gas law) allowed us to obviate one of the initial parameter dependencies. As anticipated in Section 2.3, the extraction of concentration values from the raw WTSL-DIAL data relies on the initial values that are used to compute the expected absorbances. By using a feedback scheme where a low-pass version of the measured concentration was used to compute the absorbances for the next concentration measurement, we removed the dependency on the initial guess of partial pressure, provided the system converged. The dependence on the other parameters, in particular on total pressure and OFF-resonance frequency, remained. The reliance on total pressure estimates is particularly troublesome due to the functional form of the absorbance and the strong relationship between total pressure and linewidth. In HITRAN2020, the HWHM of a transition as a function of temperature T (expressed in Kelvin), total pressure p and partial pressure $p_{p}$ (expressed in atmospheres) is modelled as

(9) $$\gamma \left(p,p_{p},T\right)={\left(\frac{296}{T}\right)}^{n}\left[\gamma_{air}\left(p-p_{p}\right)+\gamma_{self}p_{p}\right]$$

where n, $\gamma_{air}$ and $\gamma_{self}$ are tabulated transition parameters. This means that, especially in conditions where the partial pressure is low compared to the total pressure, the uncertainty and systematic error on p strongly impact the measured concentration. Moreover, using an OFF-resonance wavelength close to resonance makes the method more susceptible to pressure uncertainty, because absorbance changes more rapidly around the peak, thus leading to a larger error, according to Equation (8). Additionally, HITRAN2020 provides the $\gamma_{air}$ and $\gamma_{self}$ parameters for the 1.5711 µm transition with a relative error between 2% and 5%. Similarly to the case of the line intensity S for dTDLAS, which also applies here, the shown uncertainties correspond only to our experimental uncertainties.

**Figure 9:**
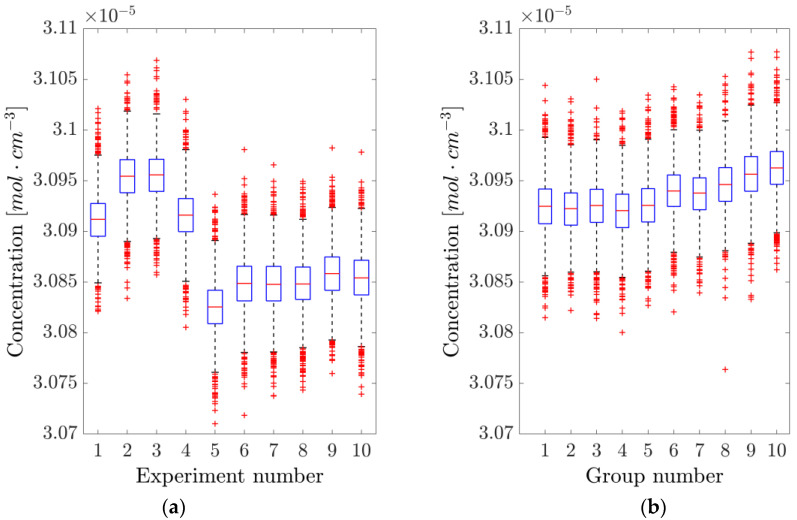
Analysis of the repeatability of the WTSL-DIAL method. (**a**) The concentration is measured by resetting the system and acquiring 1 s of data. (**b**) The system is left running, and data is acquired every 2 min. The blue boxes represent the 25th and 75th percentile, the orange horizontal lines are the medians, black whiskers represent the 99.3% confidence interval, while the red crosses indicate outliers.

As in the dTDLAS case, multiple 1 s acquisitions, each corresponding to 2000 concentration readings, were performed both with and without taking the laser below the threshold. Again, although the results are not compatible, the maximum absolute relative difference among the averages of subsequent experiments was 0.30% (mean 0.08 ± 0.03%) when resetting the initial conditions and only 0.045% (mean 0.021 ± 0.004%) when allowing the system to run without resets. These results, illustrated in Figure 9, suggest that restarting the system has a relevant effect on the dTDLAS approach, probably due to slight variations in the settling point of the hill-descent algorithm at the time of acquisition.

**Figure 10:**
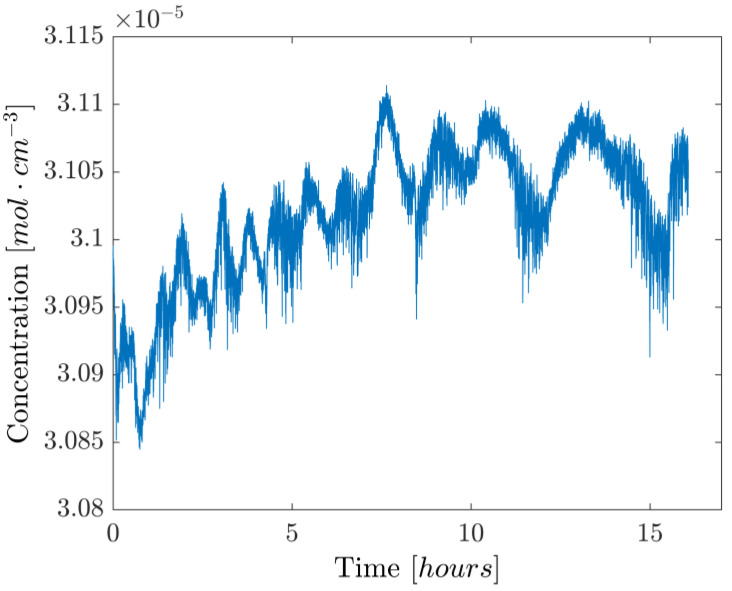
Long-term gas-sensing test with active wavelength-locking and pressure feedback loop. A decimation factor of 200 was used to reduce the amount of data.

**Figure 11:**
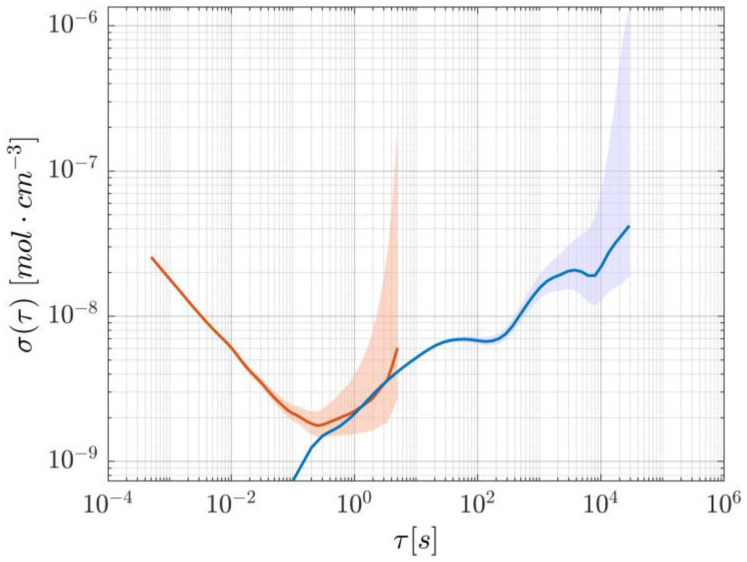
Allan deviation of the WTSL-DIAL method. The short-timescale curve, in orange, is derived from a 10 s experiment, whereas the blue long-timescale curve comes from the decimated acquisition shown in Figure 10. The shaded areas represent the respective 95% confidence intervals. For the calculation of the confidence interval on long-timescale-derived Allan deviation, a more appropriate random walk model was used in the estimation of the degrees of freedom [20].

Lastly, a ~16 h concentration measurement was performed to evaluate the stability of the setup over a protracted time interval. Due to the amount of data involved, the acquisition included a decimation factor of 200, with a passband filter between 1 and 3 Hz to avoid aliasing, so that an effective acquisition rate of 10 Hz was achieved. The resulting concentration values are shown in Figure 10. Despite having a relative peak-to-peak height of 0.87%, there are clearly identifiable oscillations that happen on longer timescales (~hour). These fluctuations appear in the Allan variation as local minima, and from Figure 11 it can be seen that their presence appears at a time scale of 100 and 10,000 s. This is in line with previous work [15] relying on a similar setup and is most likely due to the change in the room’s temperature affecting the optical properties of the optical components. In fact, forcing a change of temperature by directly exposing the components to a flow of air warmer than the room’s temperature showed a strong dependency of the beamsplitter’s properties on its actual temperature, likely due to the expansion/contraction of the dielectric coating responsible for the beam-splitting action. To suppress this effect, thermal insulation and temperature control of the whole system should be considered, while other design options, such as using an in-fiber beamsplitter and a fiber-coupled reference detector, could be explored.

The most relevant figures of merit for the WTSL-DIAL technique are reported in Table 2.

## 5. Comparison Between Methods

The main metrics used to compare the results obtained from the dTDLAS and WTSL-DIAL approaches are precision, accuracy, repeatability, stability over time and speed of data analysis. Other points of comparison are the amount of data to be processed, the readiness time after system turn-on and the complexity of operating the system.

When it comes to experimental precision, in the WTSL-DIAL approach, the datapoints were distributed around the mean value with an average relative standard deviation of 8.50 ± 0.09 ‱ during the 1 s experiments previously discussed. Equivalently, dTDLAS showed a relative standard deviation of 31.1 ± 0.1‱, which makes WTSL-DIAL 3.65 ± 0.04 times more precise than the scanning method. Unfortunately, though, the high experimental precision of both methods is overshadowed by the uncertainty on the parameters used to compute concentration values, as described by Equation (5) (dTDLAS) and Equation (6) (WTSL-DIAL). The difference between the two lies in the fact that WTSL-DIAL relies on the HWHM parameter γ, whereas in dTDLAS, one can opt for leaving the HWHM as a fitting parameter and replace γ with the time-to-wavelength conversion factor k, whose uncertainty can potentially represent an improvement over that of γ. In conclusion, while the experimental uncertainty is significantly lower when using WTSL-DIAL, the replacement of γ with k could mean a lower final uncertainty figure in the sawtooth scanning approach. In our specific case, a 2% relative uncertainty on k makes the non-experimental uncertainty comparable for both methods (2.25% for dTDLAS and 2.11% for WTSL-DIAL), but our Monte Carlo simulations also show that an exact k would reduce the dTDLAS uncertainty to 1.04%, substantially limited by the error on the main line’s intensity parameter S. Therefore, reducing the error on k by introducing wavemeters or other means of wavelength measurement (e.g., Fabry–Perot interferometers) could potentially improve the overall precision of the instrument at the expense of complexity.

In terms of accuracy, both methods have the property of being completely calibration-free thanks to the current-wavelength characterization of the laser and the feedback loop introduced in the toggling approach. Despite this, there is still a slight difference between the two methods when deriving concentration values from raw data, which makes WTSL-DIAL more prone to systematic errors: its the dependency on total pressure. In fact, pressure has a profound impact on the spectral width of the absorption lines, as shown by Equation (9). Since our setup does not encompass a highly precise barometer, we must rely on the nominal pressure values of the gas cell when calculating the $\gamma_{i}$ values needed to compute Equation (8), from which we then extract the concentration. On the other hand, the fitting procedure of the scanning approach utilizes the width of the *i* = 4 absorption line as a fitting parameter and therefore lifts the dependency on the total pressure values. Indeed, dTDLAS does not require the presence of a barometer nor knowledge of the nominal pressure value to operate. As the only other pressure dependency in this method is in the absolute position of the absorption lines, and the center of the *i* = 4 line is a fitting parameter, total pressure enters fitting only through the difference in the pressure-induced shifts in the lines’ positions. The effect of this on concentration is completely negligible compared to the uncertainty on the L, S and k parameters (refer to Equation (5)). The same is true for the partial pressure values, where a realistic guess is enough to avoid inducing relevant systematic errors. This discussion is in line with our simulation results. In summary, due to a much stronger dependency on pressure, WTSL-DIAL is more subject to systematic errors and thus generally less accurate than dTDLAS unless a pressure sensor is included. Such a sensor should not contribute significantly to the uncertainty on γ; hence, it should have a relative precision better than ~1‰.

**Figure 12:**
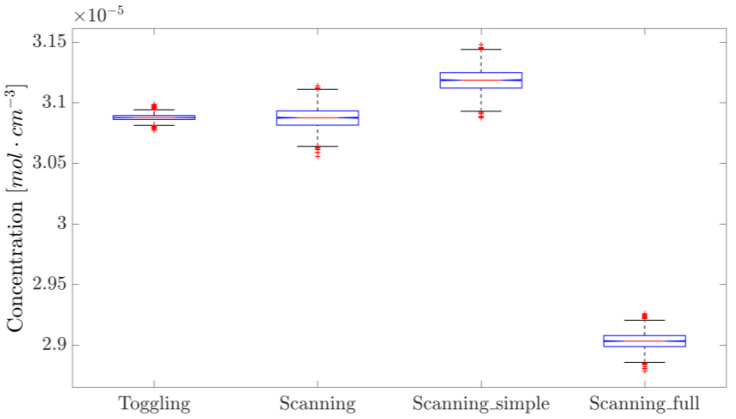
Concentration values measured with different approaches. “Scanning_simple” utilizes the *i* = 4 line only, whereas “Scanning_full” uses 6 lines and the last 60 points per period and thus does not exclude those where the wavelength response is far from linearity. These measurements were performed one after the other in the order they appear on the x-axis, except for “Scanning” and “Scanning_simple”, which originate from the analysis of the same dataset. The blue boxes represent the 25th and 75th percentile, the orange horizontal lines are the medians, black whiskers represent the 99.3% confidence interval, while the red crosses indicate outliers.

**Figure 13:**
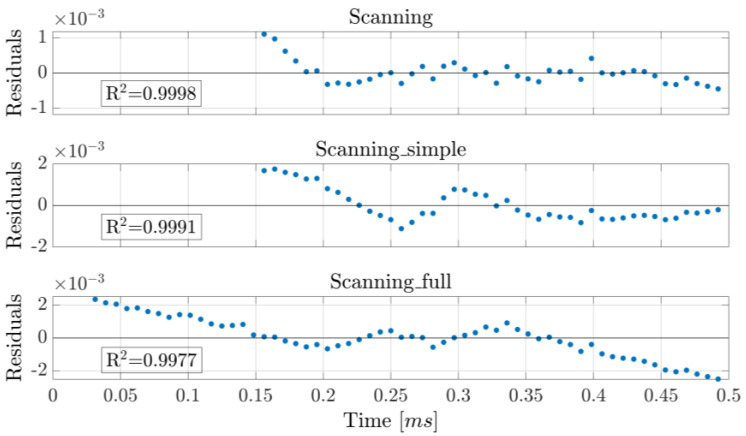
Residuals obtained from fitting the raw data used to produce Figure 4, as described in Figure 12. Only the “Scanning” approach shows normally distributed residuals and no clear trend.

The repeatability of the concentration measurements using the two approaches is illustrated in Figure 5 and Figure 9. While resetting the laser and the driving programs negatively impacts its repeatability compared to taking measurements without resets, WTSL-DIAL still outperforms dTDLAS in this metric. In particular, the maximum absolute relative excursion of subsequent concentration values observed when resetting the system was 0.30% for WTSL-DIAL and 0.37% for dTDLAS. Moreover, subsequent experiments conducted using different methods show that the difference between the extracted concentrations is within the repeatability range. This can be observed in Figure 12, where subsequent concentration measurements made using WTSL-DIAL and dTDLAS are shown side by side. This compatibility is extremely important because it supports the absoluteness of the two quantification methods when all relevant parameters, such as temperature and pressure, are known. The graph also shows what happens when the dTDLAS approach uses a single absorption line for fitting and when it considers 60 points out of the available 64 (thus including those where wavelength changes in time are not linear; see Figure 3). Figure 13 contains an example of residuals for the three models used for fitting, clearly showing that the data are better described by the multiple-absorption-line model we opted for.

**Figure 14:**
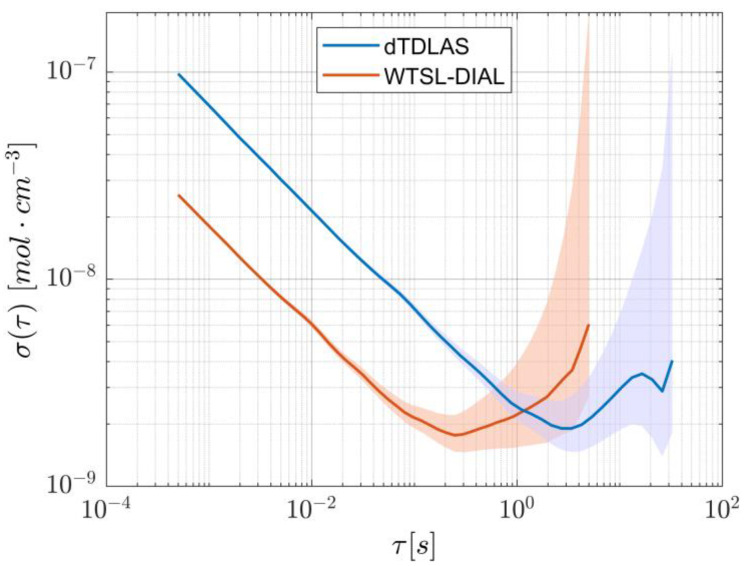
Allan deviation calculated for the two methods. Although they reach almost identical minimum values, WTSL-DIAL does so at a shorter averaging time. The shaded areas represent the respective 95% confidence intervals.

The stability of the two approaches over time can be assessed by comparing their Allan deviations, as portrayed in Figure 14. Although both methods reach approximately the same minimum values, WTSL-DIAL does so in ~0.25 s, whereas dTDLAS takes ~4 s. This means that WTSL-DIAL can be as stable as dTDLAS but operates more than one order of magnitude faster. Furthermore, the fitting procedure would limit real-time concentration measurements using the scanning approach to about 20 Hz (compared to the 2 kHz used in this paper) with our current setup, making WTSL-DIAL the only option for applications that require real-time quantification of concentrations varying on timescales smaller than the millisecond and is nonetheless much more desirable when the update rate should surpass the 1 Hz mark (crossing point of the Allan deviations). On the contrary, when speed is not a relevant metric, the scanning method is characterized by a smaller Allan deviation which, combined with superior robustness against systematic errors, makes it the more appropriate solution.

Although secondary in relevance to the experiments shown here, as well as to real-time measurements, the amount of data to be stored might be a limiting factor in some applications. In the case of data post-processing, the dTDLAS approach would require storing a whole scan per measurement which, for our setup, would translate to 44 datapoints stored at a rate of 2 kHz. Moreover, the calculation of the factor needed to convert the fit parameters to concentration, according to Equation (5), should also be stored, thus reaching 45 points to be stored per scan. On the other hand, the computational simplicity of the WTSL-DIAL approach makes it naturally suitable for real-time, high-update rate measurements, thus requiring a single value to be stored for every period. In situations where real-time processing is limited to elementary arithmetic operations, WTSL-DIAL would require two values to be stored per period, namely the ratio of the ON- and OFF-resonance averages, and the conversion factor that allows the inversion of Equation (8). In both scenarios, dTDLAS is much more demanding in terms of data storage. However, dTDLAS is faster when it comes to readiness after system start-up. In fact, its only requirement is for the laser to reach thermal equilibrium, whilst WTSL-DIAL must rely on the convergence of the hill-descent algorithm to ensure locking of the ON-resonance wavelength. For the algorithm not to increase noise significantly, the convergence rate must be kept low, which in turn extends the waiting time. This means that, with our current configuration, dTDLAS is operational roughly 30 s after startup, whereas WTSL-DIAL takes around 60 s (30 s for initial setup, 30 s for algorithm convergence). The relevance of this timing depends strongly on the specific application.

The last aspect to consider is the increased difficulty in utilizing and making the toggling system operational. Not only does it require a thorough characterization of the setup’s impulse response [15], needed to find the OFF-resonance wavelength value, but it also needs an empirical optimization of the current pulses’ shape, to minimize the transition time between wavelengths, as well as the parameters of the wavelength-locking algorithm. Fortunately, though, these are one-time procedures, which means that they only affect the installation of the system rather than its operation. If the aging of the components is relevant, then characterizations need to be repeated (for both WTSL-DIAL and dTDLAS, as they both rely on the knowledge of the laser’s current-wavelength characteristics), hence making maintenance of the systems operating the WTSL-DIAL approach more cumbersome.

## 6. Single-Detector dTDLAS

To make the results of this research comparable with other studies on the topic, especially the ones regarding the 1/f noise tolerant method called wavelength-modulation spectroscopy (WMS) [21], the performance of the single-detector dTDLAS approach was evaluated using this setup. No changes were made to the system, as the operation in this regime simply requires the analysis of the readings of the “gas arm” photodiode (PD_2_ in Figure 1). The nomenclature used for the various methods studied is the following: “1-PD scanning” is the single-detector approach, in which the baseline is fitted using a second-order polynomial (following the work in [22]); “2-PD scanning_b2” is the double detector approach, in which the baseline is fitted using a second-order polynomial; “2-PD scanning_b0” is the double detector approach, in which the baseline is fitted using a constant; and “Toggling” is the WTSL-DIAL approach discussed until now. Note that the first two methods rely on a six-parameter fit to extract concentration, namely three parameters to fit the baseline and three to fit the absorption profile determined by the six absorption lines of interest; nonetheless, their investigation is relevant, due to the observed baseline instability over time.

**Figure 15:**
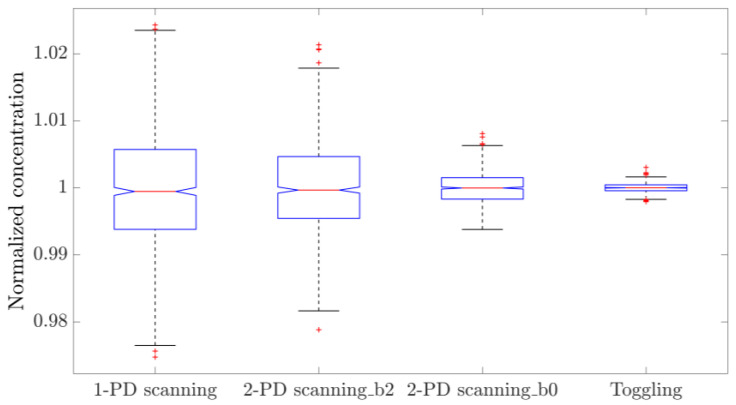
From left to right, the normalized concentrations measured with single-detector dTDLAS, two-detector dTDLAS with second-order polynomial baseline fitting, two-detector dTDLAS with flat baseline and WTSL-DIAL with partial pressure feedback. This comparison shows the relative precision of the different methods. The blue boxes represent the 25th and 75th percentile, the orange horizontal lines are the medians, black whiskers represent the 99.3% confidence interval, while the red crosses indicate outliers.

After ensuring the flatness of the baseline within the scanned region, all methods were sequentially used to acquire 1000 concentration values each. By plotting the normalized distributions, an immediate comparison of the precision of the different approaches can be made, as shown in Figure 15.

The relative standard deviations of the distributions shown in Figure 15 are summarized in Table 3. It can be concluded that the two-detector approach exhibits better precision compared to the single-detector implementation. Furthermore, ensuring the flatness of the baseline, and thus removing two parameters from the fitting algorithm, makes the method much more precise; however, this requirement cannot be guaranteed over time if the entire system is not thermostatic, due to the temperature dependencies of the various components. Finally, WTSL-DIAL is still the most precise method, showing a substantial improvement by a factor of 12.7 ± 0.4 over the single-detector dTDLAS; this means that more than 160 single-detector scans are required to match the WTSL-DIAL method’s precision. This is reminiscent of the precision improvement of WMS over single-detector dTDLAS demonstrated in a previous study [21].

## 7. Conclusions

Direct-mode tunable diode-laser absorption spectroscopy (dTDLAS) and wavelength-toggled single-laser differential absorption lidar (WTSL-DIAL), two laser-based remote sensing techniques for gas quantification, were compared using the same two-detector system operating at 2 kHz. The semi-empirical models used to extract the concentrations from transmittance measurements were analyzed, showing how dTDLAS can be operated without the need for accurate barometers. On the contrary, WTSL-DIAL relies more on the estimated parameters, especially total and partial pressure values, although the latter was mitigated by introducing a feedback approach that exploits WTSL-DIAL’s ability to provide real-time measurements. Moreover, the theoretical background of WTSL-DIAL was expanded to include the effect of the signal’s periodicity in the calculation of the OFF-resonance wavelength, thus estimating it more accurately. In fact, the main advantage of WTSL-DIAL is that it is fully arithmetic, and no fitting operations are required, making it much faster and apt for fast update rates and real-time gas quantification.

In terms of precision, the experimental uncertainty of WTSL-DIAL is 3.65 ± 0.04 times smaller than the one achieved with two-detector dTDLAS and 12.7 ± 0.4 times better than the single-detector dTDLAS approach. Despite this, the total uncertainty on the measurements is dominated by the parameters used to calculate the multiplicative factor that links absorbance to concentration, according to Equation (5) (dTDLAS) and Equation (6) (WTSL-DIAL). The difference between the two methods can be explained as follows: the toggling approach relies on the HWHM parameter γ, whose uncertainty is determined by the library used to retrieve the line parameters. In contrast, when using the scanning approach, one can treat the HWHM as a fitting parameter and replace γ with the time-to-wavelength conversion factor k, whose uncertainty can potentially represent an improvement over that of γ. In our case, such non-experimental uncertainty is 2.25% for dTDLAS and 2.11% for WTSL-DIAL; however, Monte Carlo simulations show that the dTDLAS value could be significantly improved by reducing the uncertainty on k.

On top of the improved experimental precision over dTDLAS, WTSL-DIAL also performed better in terms of repeatability, with a 0.30% maximum absolute relative difference between subsequent experiments (mean 0.08 ± 0.03%) compared to the 0.37% (mean 0.10 ± 0.04%) of dTDLAS. In terms of accuracy, although not compatible, the two methods yielded results that remained within each other’s repeatability range, but WTSL-DIAL was less robust against systematic errors, particularly the ones involving pressure.

Lastly, comparing the two-detector methods against the single-detector dTDLAS paradigm revealed that using two detectors reduces noise by a factor 1.31 ± 0.04 at 2 kHz when a second order polynomial is used for baseline fitting, whereas experimentally ensuring the flatness of the baseline and fitting it with a constant increases this figure to 3.7 ± 0.1. Although this represents an improvement, its relatively low magnitude makes it worth considering whether introducing a second detector (and potentially other optical components) is preferable when applying dTDLAS at 2 kHz.

## Figures and Tables

**Table 1:** Summary of the dTDLAS method results.

Reproducibility with system restart [%]	0.10 ± 0.04 (max. 0.37)
Reproducibility without system restart [%]	0.08 ± 0.04 (max. 0.42)
Minimum Allan deviation [mol·cm^−3^]	1.85 × 10^−9^ (at 4.1 s)

**Table 2:** Summary of the WTSL-DIAL method results. The minimum Allan deviation corresponds to the minimum value calculated using the short-timescale data shown in Figure 11.

Reproducibility with system restart [%]	0.08 ± 0.03 (max. 0.30)
Reproducibility without system restart [%]	0.021 ± 0.004 (max. 0.045)
Minimum Allan deviation [mol·cm^−3^]	1.76 × 10^−9^ (at 0.26 s)

**Table 3:** Standard error of the normalized concentration distributions measured with the different methods studied.

Method	Relative Standard Error [‰]
1-PD scanning	8.8 ± 0.2
2-PD scanning_b2	6.7 ± 0.2
2-PD scanning_b0	2.4 ± 0.1
Toggling	0.70 ± 0.02

## Data Availability

Data underlying the results presented in this paper are available in Ref. [23].

## Supplementary Materials

The following supporting information can be downloaded at: https://www.mdpi.com/article/10.3390/s25196064/s1.
