# Supplementary material for: Benchmarking Precompensated Current-Modulated Diode-Laser-Based Differential Absorption Lidar for CO2 Gas Concentration Measurements at kHz Rate
Source: Sensors (Basel). 2025 Oct 2;25(19):6064. doi: 10.3390/s25196064 (PMC12526940; doi:10.3390/s25196064)
Supplement: Supplementary file 1 [file sensors-25-06064-s001.zip › sensors-3841510-supplementary.pdf]

Supplementary material for

Benchmarking precompensated current-modulated diode laser based differential absorption lidar for CO_2 gas concentration measurements at kHz rate

GIACOMO ZANETTI*, PETER JOHN RODRIGO, HENNING E. LARSEN, AND CHRISTIAN PEDERSEN

DTU Electro, Department of Electrical and Photonics Engineering, Technical University of Denmark, Frederiksborgvej 399, Building 128, 4000 Roskilde, Denmark
**giaza@dtu.dk*

Modelling of the laser's periodic wavelength tuning

The laser was modeled as a time-invariant system able to linearly translate applied currents above the lasing threshold into wavelength modulations. Under these assumptions, an impulse response $h(t)$ can be defined, and the wavelength modulation in time $u(t)$ is related to the input current above threshold $I(t)$ as

$$u(t) = (h * I)(t) \quad \text{(S1)}$$

where * denotes the convolution operation. As is customary, this type of equation is handled using the Laplace transform formalism, so that Eq. S1 becomes

$$\tilde{u}_{(s)} = \tilde{h}_{(s)}\tilde{I}_{(s)} \quad \text{(S2)}$$

with the ˜ indicating the Laplace transform of a function.
For a periodic function f with period T, the Laplace transform is defined as

$$\tilde{f}_{(s)} = \frac{1}{1 - e^{-st}}\tilde{f}_p(s) \quad \text{(S3)}$$

where $\tilde{f}_p$ is the Laplace transform of f calculated over a single period.

Practically, to measure the impulse response of the system, a periodic train of square pulses was applied (2 kHz frequency, 50 % duty cycle) and the wavelength modulation in time was measured. In steady-state conditions, both the input and output signals have the same frequency, hence the impulse response can be defined as the ratio between the single-period Laplace transforms thanks to the previous equations, namely as

$$\tilde{h}_{(s)} = \frac{\tilde{u}_p(s)}{\tilde{I}_p(s)} \quad \text{(S4)}$$

Given that $I(t)$ is a period train of square pulses with amplitude I_0, period $T = \frac{1}{2000\,Hz}$ and 50 % duty cycle, its single-period Laplace transform is

$$\tilde{I}_p(s) = \int_0^{T/2} I_0\, e^{-st} dt = \frac{I_0}{s}\left(1 - e^{-\frac{sT}{2}}\right) \quad \text{(S5)}$$

which, combined with Eq. S4, means that the impulse response in the complex frequency domain can be expressed as

$$\tilde{h}_{(s)} = \frac{s\tilde{u}_p(s)}{I_0\left(1 - e^{-\frac{sT}{2}}\right)} \quad \text{(S6)}$$

where we notice that $\tilde{u}_p$ is the single-period Laplace transform of the measured wavelength change in time.

1. dTDLAS

In dTDLAS, the applied current has a sawtooth profile, meaning that it is a linear ramp over one period. This means that $\tilde{I}_p(s)$ is

$$\tilde{I}_p(s) = \frac{I_{max}}{T'}\int_0^{T'} t\, e^{-st} dt = \frac{I_{max}}{s^2 T'}\left(1 - e^{-sT'} - sT' e^{-sT'}\right) \quad \text{(S7)}$$

Where I_{max} is the maximum applied current, T' is the periodicity of the ramp, which can in principle be different from the period T used to characterize the impulse response of the system. The wavelength response over one period T' is found as

$$\tilde{u}'_p(s) = \tilde{h}_{(s)}\tilde{I}_p(s) = \frac{I_{max}}{I_0 T'}\frac{\tilde{u}_p(s)}{s}\frac{1 - e^{-sT'}}{1 - e^{-\frac{sT}{2}}} - \frac{I_{max}}{I_0}\frac{\tilde{u}_p(s)}{1 - e^{-\frac{sT}{2}}}e^{-sT'} \quad \text{(S8)}$$

Now, if we drive the laser at double the frequency of the one used for characterization, namely so that $2T' = T$, the previous equation becomes

$$\tilde{u}'_p(s) = \tilde{h}_{(s)}\tilde{I}_p(s) = \frac{I_{max}}{I_0}\left[\frac{\tilde{u}_p(s)}{sT'} - \tilde{u}_p(s)e^{-sT'} - \frac{\tilde{u}_p(s)e^{-2sT'}}{1 - e^{-sT'}}\right] \quad \text{(S9)}$$

Since $t \in [0, T']$ and the exponentials in the complex frequency domain act as a time-delay in the time domain, the wavelength response is calculated as

$$u'_p(t) = L^{-1}\left[\frac{I_{max}}{I_0}\frac{\tilde{u}_p(s)}{sT'}\right](t) = \frac{I_{max}}{I_0 T'}\int_0^t u_p(\tau)d\tau \quad \text{(S10)}$$

where L^{-1} represents the inverse Laplace transform operator and $u(0) = 0$.
Again, applying this would require the knowledge of the impulse response measured at half the frequency of operation of the laser. In reality, we assumed that the wavelength response at a frequency $f = \frac{1}{T}$ and at $f' = \frac{1}{T'}$ would be indistinguishable for the first T', hence requiring the knowledge of the impulse response at 2 kHz only.

2. WTSL-DIAL

In this case, the applied current over one period has the exponential form

$$I_p(t) = \begin{cases} I_{box} + I_{exp} e^{-\frac{t}{\tau}}, & t < \frac{T'}{2} \\ -I_{exp} e^{-\frac{\left(t-\frac{T'}{2}\right)}{\tau}}, & t \geq \frac{T'}{2} \end{cases} \quad \text{(S11)}$$

where I_{box}, I_{exp} and τ are experimentally set constants, while T' is the period of the modulation. This makes the single-period Laplace transform of the current above threshold be

$$\tilde{I}_p(s) = \frac{I_{box}}{s}\left(1 - e^{-\frac{sT'}{2}}\right) + \frac{I_{exp}}{s + \frac{1}{\tau}}\left(1 - e^{-\frac{sT'}{2}}\right)\left(1 - e^{-\frac{sT'}{2}} e^{-\frac{T'}{2\tau}}\right) \quad \text{(S12)}$$

Now, the impulse response calculated as per Eq. S6 can be multiplied to $\tilde{I}_p(s)$ to obtain the wavelength response. We notice that $\tilde{I}_p(s)$ contains the term $\left(1 - e^{-\frac{sT'}{2}}\right)$ in the numerator, and that cancels out with the one in the denominator of $\tilde{h}_{(s)}$ when the two are multiplied together. This leaves us with the wavelength response

$$\begin{aligned} \tilde{u}'_p(s) = \tilde{h}_{(s)}\tilde{I}_p(s) &= \frac{I_{box}}{I_0}\tilde{u}_p(s) + \frac{I_{exp}}{I_0}\frac{s}{s + \frac{1}{\tau}}\left(1 - e^{-\frac{sT'}{2}} e^{-\frac{T'}{2\tau}}\right)\tilde{u}_p(s) \\ &= \frac{I_{box}}{I_0}\tilde{u}_p(s) + \frac{I_{exp}}{I_0}\left(1 - \frac{1}{1 + s\tau}\right)\left(1 - e^{-\frac{sT'}{2}} e^{-\frac{T'}{2\tau}}\right)\tilde{u}_p(s) \end{aligned} \quad \text{(S13)}$$

If we define an auxiliary periodic function $x(t)$, with period T', so that over one period

$$x_p(t) = \begin{cases} \frac{e^{-\frac{t}{\tau}}}{\tau}, & t < \frac{T'}{2} \\ 0, & t \geq \frac{T'}{2} \end{cases} \quad \text{(S14)}$$

we can see that its single-period Laplace transform is

$$\tilde{x}_p(s) = \frac{1}{1 + s\tau}\left(1 - e^{-\frac{sT'}{2}} e^{-\frac{T'}{2\tau}}\right) \quad \text{(S15)}$$

Using Eq. S15 we can rewrite Eq. S13 as

$$\tilde{u}'_p(s) = \left(\frac{I_{box}}{I_0} + \frac{I_{exp}}{I_0} - \frac{I_{exp}}{I_0} e^{-\frac{sT'}{2}} e^{-\frac{T'}{2\tau}}\right)\tilde{u}_p(s) - \frac{I_{exp}}{I_0}\tilde{x}_p(s)\tilde{u}_p(s) \quad \text{(S16)}$$

Therefore, by exploiting the behavior of the convolution under the Laplace transform formalism we can write the wavelength response in time as

$$u'_p(t) = \left(\frac{I_{box}}{I_0} + \frac{I_{exp}}{I_0}\right) u_p(t)\vartheta(t) - \frac{I_{exp}}{I_0} e^{-\frac{T'}{2\tau}} u_p\left(t - \frac{T'}{2}\right)\vartheta\left(t - \frac{T'}{2}\right) - \frac{I_{exp}}{I_0}\left(x_p(t) * u_p(t)\right)\vartheta(t) \tag{S17}$$

which can be readily calculated knowing that $t \in [0, T']$ and $\vartheta(t)$ is the Heaviside function.
